# Determining Minimum Trial Numbers for Reliable Lameness Detection in Canine Kinematic Studies

**DOI:** 10.3390/ani16040624

**Published:** 2026-02-16

**Authors:** Isabel Marrero, Angelo Santana, José Manuel Vilar

**Affiliations:** 1 Hospital Clinico Veterinario, Universidad de Las Palmas de Gran Canaria, Trasmontaña S/N, 35416 Arucas, Las Palmas, Spain; isabel.marrero@fpct.ulpgc.es; 2 Departamento de Matemáticas, Universidad de Las Palmas de Gran Canaria, 35018 Las Palmas de Gran Canaria, Las Palmas, Spain; angelo.santana@ulpgc.es; 3 Instituto Universitario de Investigaciones Biomédicas y Sanitarias, Universidad de Las Palmas de Gran Canaria, Trasmontaña S/N, 35416 Arucas, Las Palmas, Spain

**Keywords:** gait analysis, symmetry indices, delta method, trial number, lameness

## Abstract

Visual orthopedic gait assessment in dogs is recognized as subjective and is limited by interobserver variability. Objective detection of lameness is offered by biomechanical analysis, where asymmetry between limbs is quantified through kinematic parameters and symmetry indices. However, the minimum number of trials (full stride cycles) required to reliably discriminate lameness has remained a challenge. In this study, six healthy adult dogs were used. Mild, reversible lameness was induced in one forelimb using a cotton pad. Dogs were walked along a straight runway, and kinematic data were captured with a high-speed video camera. Stride length (SLE), support time (ST), and elbow range of motion (ROM) were measured. Symmetry indices (for linear and temporal parameters) and the symmetry angle (for angular parameters) were computed. The asymptotic distribution of these indices was derived using the delta method, which allowed for the construction of confidence intervals (CIs) and hypothesis tests for an asymmetry threshold of 3%. The number of trials required to achieve reliable detection was estimated through statistical simulations. Results indicated that the required number of trials was highly dependent on both the kinematic parameter and the magnitude of asymmetry. While detecting subtle asymmetries (≈4%) required a high number of trials (up to 347 for stride length), the requirements decreased substantially for more pronounced lameness. For a true asymmetry of 6%, 11–39 trials per limb were sufficient to achieve 80–90% power. It is concluded that the collection of only five trials is insufficient for detecting mild asymmetries. A statistical framework and practical recommendations for kinematic gait studies in dogs are provided.

## Simple Summary

Visual gait assessment in dogs is considered subjective and is often associated with inconsistency, especially when lameness is subtle. To address this, biomechanical analysis provides objective measures through the comparison of kinematic variables between limbs. However, the collection of an insufficient number of trials can lead to unreliable conclusions, while the collection of too many is inefficient and may result in the unnecessary use of more animals than required for a specific research objective. In this pilot study, mild forelimb lameness was induced in six dogs, and their gait was filmed with a high-speed camera. A statistical framework based on the delta method was employed to determine the number of trials (i.e., full stride cycles) required to distinguish lameness from normal variability. It was demonstrated that the commonly used five trials are insufficient. Instead, the required number of trials is highly parameter-specific. Depending on the severity of lameness and the kinematic variable measured, reliable detection could require from under 20 to over 300 trials per limb. These findings provide practical guidelines for both clinical and research settings to improve the accuracy of lameness detection.

## 1. Introduction

Visual orthopedic gait assessment is highly subjective because it depends on the observer’s skill level; therefore, it is prone to interobserver variability; this subjectivity is particularly problematic when lameness is subtle or bilateral [1,2,3].

Biomechanical analysis offers an objective alternative by quantifying gait-related parameters, allowing the detection of lameness when differences in biomechanical values are observed between limbs [2]. This often involves inducing harmless, reversible lameness in canine models [4,5,6] and calculating symmetry indices (SIs) or the symmetry angle (SA), where an asymmetry value between contralateral limbs greater than 3–5% is typically considered indicative of lameness [7,8,9,10,11,12]. The SI is defined as

$$SI=\frac{\left(SL-LL\right)}{\frac{1}{2}\left(SL+LL\right)}\cdot 100$$

where the terms refer to the values obtained from one limb and the corresponding contralateral limb; in this case, SL refers to the value for the sound limb (higher) and LL is the value for the lame limb (lower); as noted above, SI = 0% indicates complete symmetry and SI = 100% indicates maximal asymmetry [13,14]. On the other hand, the formula for SA [15] is calculated as

$$SA=\frac{45{}^{\circ}-arctan\left(\frac{{ROM}_{LL}}{{ROM}_{SL}}\right)}{90{}^{\circ}}\cdot 100$$

However, a critical methodological challenge remains: how to determine the minimum number of gait trials required to obtain reliable data capable of consistently distinguishing a “lame” from a “sound” condition. Traditional methods, such as the sequential average or the intraclass correlation coefficient (ICC), are often used to assess reliability [16,17,18] but have demonstrated limitations in establishing a valid, representative number of trials and are frequently inconsistent with each other [19]. A key methodological question is how to derive a single symmetry measure from multiple trials. Two approaches have been proposed: computing the mean of the index calculated for each individual trial [20] or calculating the index once using the mean values of all recorded trials as input [10,21].

Furthermore, the statistical properties of these symmetry indices are often overlooked. Previous studies apply SIs and SA without thoroughly examining their distributions, omitting inferential statistics like confidence intervals (CIs), and lacking an established method for hypothesis testing [22]. This neglect is significant given that these indices are non-linear and approach asymptotic limits under real conditions [14], making their statistical treatment non-trivial.

Consequently, there is no consensus on the number of trials (i.e., strides) needed, with most kinematic studies arbitrarily reporting 3–5 valid trials [14,23]. This practice is problematic, as a single trial (stride) is insufficient for reliable extrapolation [24,25], and an inadequate sample size can lead to underpowered studies that fail to detect subtle lameness or, conversely, to inefficient data collection [26,27]. Although current motion capture systems allow for the acquisition of a large number of trials under standardized conditions with relative ease, such technology is not commonly available in clinical or sports settings due to practical and economic constraints. Therefore, establishing the minimum number of valid kinematic trials required to reliably detect low-grade lameness is of particular importance.

To address this gap, a robust statistical framework for determining sample size in kinematic studies is required. The delta method is a powerful statistical tool used to derive the asymptotic distribution of functions of estimated parameters, making it ideally suited for analyzing the distribution of non-linear symmetry indices. Therefore, after verifying that the appropriate conditions for its application are met, the primary objective of this study was to apply the delta method to derive the asymptotic distribution of commonly used symmetry indices (SI and SA) and to utilize this framework to provide practical guidelines for constructing CIs, performing hypothesis tests, and, most importantly, calculating the sample sizes needed to detect mild unilateral forelimb lameness in dogs at a common asymmetry threshold (≈3%).

## 2. Materials and Methods

### 2.1. Animals

Six healthy, medium-sized adult dogs participated in the study. The animals were privately owned dogs belonging to clients of the Veterinary Teaching Hospital and Jaira Veterinary Clinic (Las Palmas de Gran Canaria, Spain); all dogs were clinically healthy and showed no signs of orthopedic or neurological disease at the time of the study. Each animal’s body condition score (BCS, 1–9) was assessed for suitability. Table 1 summarizes individual data.

### 2.2. Lameness Induction Model

Mild, reversible lameness was created by taping a small cotton pad to the metacarpal cushion of the right forelimb. The pad was secured with a strip of tape. This induced slight discomfort without injury.

### 2.3. Data Acquisition

An orthogonal reference system was created for reliable kinematic measurements.

Four purpose-built polystyrene 10 mm retroreflective markers were attached to the skin over the following palpable bony landmarks using double-sided adhesive tape and further secured with a flexible, hypoallergenic adhesive film (3M, Madrid, Spain) to minimize motion artifacts: the greater tubercle of the humerus, the lateral humeral epicondyle, the lateral aspect of the ulnar carpal bone and the head of the fifth metacarpal. The first three markers were used to derive elbow ROM (peak extension minus peak flexion); the fourth marker captured stride length.

Dogs were walked on a leash by their owners along a straight runway in both directions. For each parameter, 120 valid trials (10 trials × 2 limbs × 6 dogs) were recorded. A trial was considered valid if the dog completed a full stride cycle (defined as the interval from the placement of a forelimb to the subsequent placement of the same forelimb) while walking in a straight line at an approximately constant speed, estimated from the time required for a fixed anatomical landmark (tail base) to traverse the orthogonal coordinate system, and excluding strides with acceleration or deceleration (1.2 ± 0.2 m/s) without stopping, hesitating, or exhibiting head or body movements.

**Figure 1:**
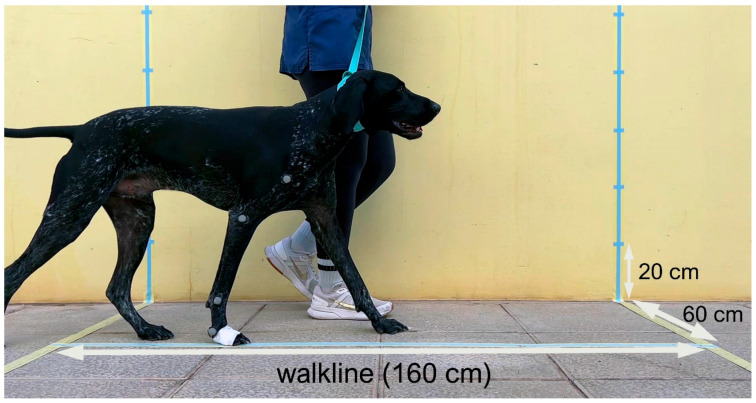
Frame corresponding to the video recording of a trial. In this case, the “lame” limb is closer to the video camera.

Gait was recorded using a high-speed video camera (120 frames/s) positioned 140 cm perpendicular to the runway. Each frame corresponded to 8.3 ms. All video footage was analyzed frame by frame to extract the kinematic variables (see Figure 1).

### 2.4. Data Processing and Outcome Variables

The raw video sequences were analyzed frame-by-frame using Kinovea® version 0.9.5 (kinovea.org) to extract the two-dimensional coordinates of the reflective markers. The following kinematic parameters were calculated for each valid stride:

Stride Length (SLE): The distance (in centimeters) between two consecutive placements of the same paw on the ground. This parameter was normalized to the animal’s body length and expressed as a percentage (SLE%).

Support Time (ST): The duration (in milliseconds) of ground contact of the paw during a stride. This parameter was normalized to the total stride time and expressed as a percentage (ST%).

Elbow Range of Motion (ROM): The angular excursion (in degrees) between full flexion and full extension of the elbow joint. The angle was calculated from the markers placed on the greater tubercle of the humerus, the lateral humeral epicondyle, and the head of the fifth metacarpal.

For the statistical analysis, the mean values of all valid trials for each dog and limb were used as the input data for the calculation of the symmetry indices.

### 2.5. Statistical Analysis

Analyses were performed in R (version 4.5.1) [28]. Custom functions computed symmetry indices (SIs), applied the delta method and produced bootstrap intervals in order to verify which method was more appropriate. Data manipulation used dplyr and purrr (version 1.2.0.); figures were generated with ggplot2 (version 4.0.5.).

#### 2.5.1. Statistical Framework and the Delta Method

To provide a robust statistical justification for our sample size calculations, we derived the asymptotic distribution of the symmetry indices using the delta method. This approach allows us to construct CIs and hypothesis tests for the asymmetry threshold.

Preliminary inspection suggested that SLE%, ST% and ROM were approximately normally distributed for each dog. Thus, measurements from the sound limb (X_S_) were modeled as $N\left(\mu_{S},\sigma_{S}\right)$ and those from the lame limb (X_L_) as $N\left(\mu_{L},\sigma_{L}\right)$. Because trials were independent, the sample means are distributed as ${\bar{X}}_{S}∼N\left(\mu_{S},\frac{\sigma_{S}}{\sqrt{n_{S}}}\right)$, and ${\bar{X}}_{L}∼N\left(\mu_{L},\frac{\sigma_{L}}{\sqrt{n_{L}}}\right)$, where n_S_ and n_L_ are the number of trials of the sound and lame limbs respectively. Even if the underlying distributions were not strictly normal, the central limit theorem ensures that sample means are asymptotically normal [29]. Independence between limbs implies a diagonal covariance matrix of the vector $\bar{X}=\left({\bar{X}}_{S},{\bar{X}}_{L}\right)$, which is $\Sigma =\left(\begin{array}{cc} \sigma_{S}^{2}/n_{S} & 0\\ 0 & \sigma_{L}^{2}/n_{L} \end{array}\right)$.

This vector converges in probability to $\mu =\left(\mu_{S},\mu_{L}\right)$. Under these conditions, if $g\left(x,y\right)$ is a continuously differentiable function, the delta method [30], ensures that $g\left(\bar{X}\right)$ converges asymptotically to

$$N\left(g\left(\mu \right),\nabla g{\left(\mu \right)}^{⊤}\Sigma \nabla g\left(\mu \right)\right)$$

where ∇g is the gradient of g at μ.

#### 2.5.2. Application to Symmetry Indices (SI and SA)

For linear or temporal parameters, we consider the function $g\left(x,y\right)=200\frac{x-y}{x+y}$. Therefore, $SI=g\left({\bar{X}}_{S},{\bar{X}}_{L}\right)$. The delta method gives

$$SI\overset{d}{\to }N\left(\mu_{SI},\sigma_{SI}\right)$$

where

$$\mu_{SI}=200\frac{\mu_{S}-\mu_{L}}{\mu_{S}+\mu_{L}},\;\sigma_{SI}=\frac{400}{{\left(\mu_{S}+\mu_{L}\right)}^{2}}\sqrt{\frac{\mu_{S}\sigma_{L}^{2}}{n_{L}}+\frac{\mu_{L}\sigma_{S}^{2}}{n_{S}}}$$

Defining the true asymmetry $A=SI=200\cdot \frac{\mu_{S}-\mu_{L}}{\mu_{S}+\mu_{L}}$ and the coefficients of variation $CV_{S}=\frac{\sigma_{S}}{\mu_{S}}$ and $CV_{L}=\frac{\sigma_{L}}{\mu_{L}}$, the distribution simplifies to $SI\overset{d}{\to }N\left(A,\sigma_{SI}\right)$, where $\sigma_{SI}=\frac{\left(200^{2}-A^{2}\right)}{400}\sqrt{\frac{CV_{S}^{2}}{n_{S}}+\frac{CV_{L}^{2}}{n_{L}}}$.

The standard error diminishes as sample size increases and grows with variability; larger asymmetry slightly reduces the error but has a minor effect within the typical clinical range.

For angular variables, we consider the function $g\left(x,y\right)=\frac{200}{\pi }\left(\frac{\pi }{4}-arctan\left(\frac{x}{y}\right)\right)$. When x and y are the mean ROMs of the lame and sound limbs we obtain $SA=g\left({\bar{X}}_{L},{\bar{X}}_{S}\right)$. The delta method yields

$$SA\overset{d}{\to }N\left(\frac{200}{\pi }\cdot \left(\frac{\pi }{4}-arctan\left(\frac{\mu_{L}}{\mu_{S}}\right)\right),\frac{200}{\pi \left(\mu_{S}^{2}+\mu_{L}^{2}\right)}\cdot \sqrt{\frac{\mu_{L}^{2}\sigma_{S}^{2}}{n_{S}}+\frac{\mu_{S}^{2}\sigma_{L}^{2}}{n_{L}}}\right)$$

With $A=\frac{200}{\pi }\cdot \left(\frac{\pi }{4}-arctan\left(\frac{\mu_{L}}{\mu_{S}}\right)\right)$, and $\mu_{L}=\mu_{S}\cdot tan\left(\pi /4-A/200\right)$, one obtains $SA\overset{d}{\to }N\left(A,\sigma_{SA}\right)$, where

$$\sigma_{SA}=\frac{100}{\pi }\cdot cos\left(\frac{\pi A}{100}\right)\cdot \sqrt{\frac{CV_{S}^{2}}{n_{S}}+\frac{CV_{L}^{2}}{n_{L}}}$$

As with SI, the standard error decreases with larger samples and increases with variability; smaller asymmetries yield larger errors due to the cosine term.

#### 2.5.3. Validation with Simulations

**Figure 2:**
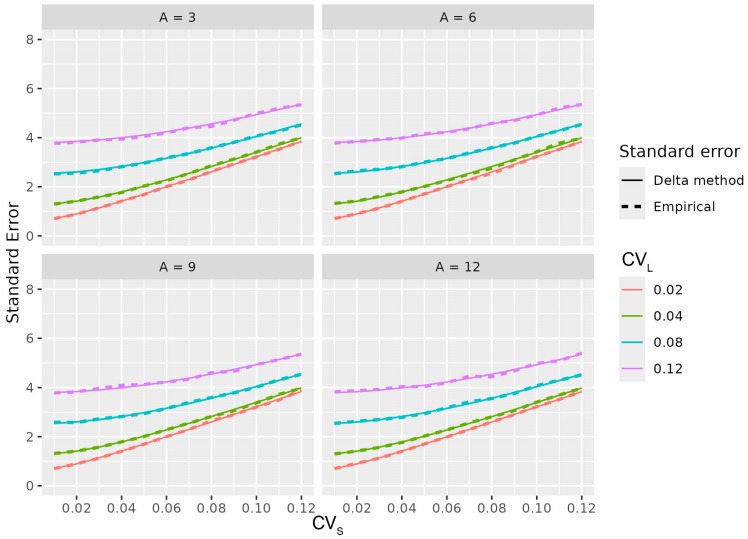
Validation of the delta-method approximation. Empirical and theoretical standard errors of the symmetry indices (SI and SA) are shown for increasing sample sizes per limb.

The delta method provides an asymptotic approximation to the sampling distribution of the estimated asymmetry index. Consequently, its accuracy may deteriorate when sample sizes are small, leading to biased or imprecise approximations to the true distribution. For this reason, simulations were conducted to verify the adequacy of the delta method approximation across a range of sample sizes. Mean values of $\mu_{S}$ and $\mu_{L}$ were fixed to produce asymmetry values A between 0 and 12. CV_S_ and CV_L_ ranged from 0.01 to 0.12. For each combination, 5000 datasets were simulated with *n* = 5, 10, 15, 20, 25 or 30 measurements per limb. Empirical means and standard errors of SI and SA agreed closely with theoretical predictions, even for *n* = 10. The Shapiro–Wilk test supported approximate normality. These findings confirm that the delta method provides accurate approximations for SI and SA, with sample sizes as small as ten trials. (Figure 2).

**Figure 3:**
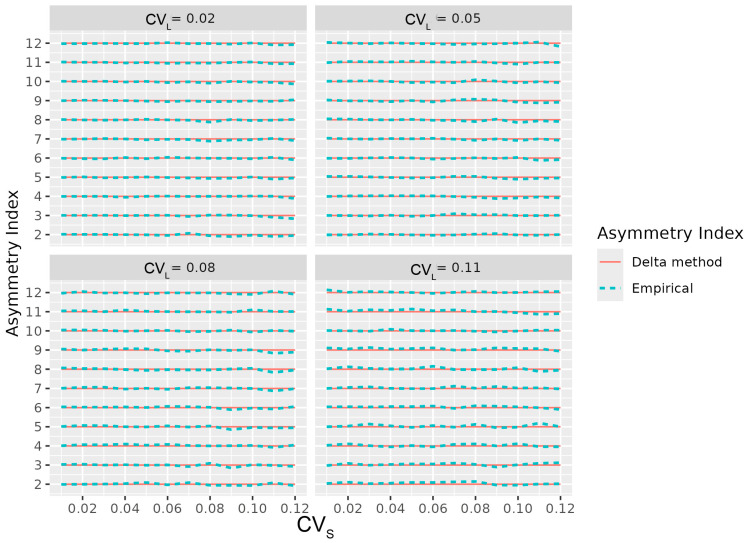
Comparison between simulated and analytical expected values of the asymmetry indices across varying asymmetry levels and coefficients of variation.

Furthermore, the simulated means of the asymmetry indices showed no systematic bias and agreed closely with the theoretical expected values across a wide range of conditions, confirming the accuracy of the delta method’s approximation (Figure 3).

#### 2.5.4. Statistical Inference: CIs and Hypothesis Testing

CIs A(1−α) for the true asymmetry A is $A\in \left[\hat{A}\pm t_{n_{S}+n_{L}-2,1-\alpha /2}{\hat{\sigma }}_{\hat{A}}\right]$, where $t_{n_{S}+n_{L}-2,1-\alpha /2}$ is the relevant quantile of Student’s t-distribution and ${\hat{\sigma }}_{\hat{A}}$ is the estimated standard error of $\hat{A}$. Simulations showed that using a t-distribution yields more accurate coverage than a normal z-score when samples are small; bootstrap methods offered no clear advantage, and thus they were no longer applied.

Lameness was declared if asymmetry exceeded 3%. The hypotheses H_0_: A ≤ 3% vs. H_1_: A > 3% were tested using $\tau =\frac{\hat{A}-3}{{\hat{\sigma }}_{\hat{A}}}>t_{n_{S}+n_{L}-2,1-\alpha }\;,\;$ compared against the t-distribution threshold. Alternatively, a *p*-value was obtained as $p=Pr\left(t_{n_{S}+n_{L}-2}>\frac{\hat{A}-3}{{\hat{\sigma }}_{\hat{A}}}\right)$, and H_0_ was rejected if *p* < α.

#### 2.5.5. Power and Sample-Size Calculation

Assuming equal measurements per limb ($n_{S}=n_{L}=n$), the standard error simplifies to

$${\hat{\sigma }}_{\hat{A}}=\left\{\begin{array}{ll} \frac{1}{\sqrt{n}}\cdot \frac{\left(200^{2}-{\hat{A}}^{2}\right)}{400}\cdot \sqrt{{\hat{CV}}_{S}^{2}+{\hat{CV}}_{L}^{2}} & =\frac{1}{\sqrt{n}}\cdot \vartheta_{\hat{SI}}\;,\;\;\;\mathrm{if}\;\hat{A}=\hat{SI}\\ \frac{1}{\sqrt{n}}\cdot \frac{100}{\pi }\cdot cos\left(\frac{\pi \hat{A}}{100}\right)\cdot \sqrt{{\hat{CV}}_{S}^{2}+{\hat{CV}}_{L}^{2}} & =\frac{1}{\sqrt{n}}\cdot \vartheta_{\hat{SA}}\;,\;\;\;\mathrm{if}\;\hat{A}=\hat{SA} \end{array}\right.$$

where ϑ$\hat{A}$ depends on the chosen index. To achieve power 1 − β at significance level α for detecting an asymmetry of 3 + Δ, the required sample size is $n≅\frac{{\left(z_{1-\alpha }+z_{1-\beta }\right)}^{2}{\hat{\theta }}_{\hat{A}}^{2}}{\Delta^{2}}$.

## 3. Results

### 3.1. Kinematic Parameters and Induced Asymmetry

The kinematic parameters for each dog and limb are summarized in Table 2. The induction of lameness resulted in significant reductions (*p* < 0.05) in SLE%, ST% and ROM in the affected limb compared to the sound limb across all subjects.

### 3.2. Data Distribution and Variability

**Figure 4:**
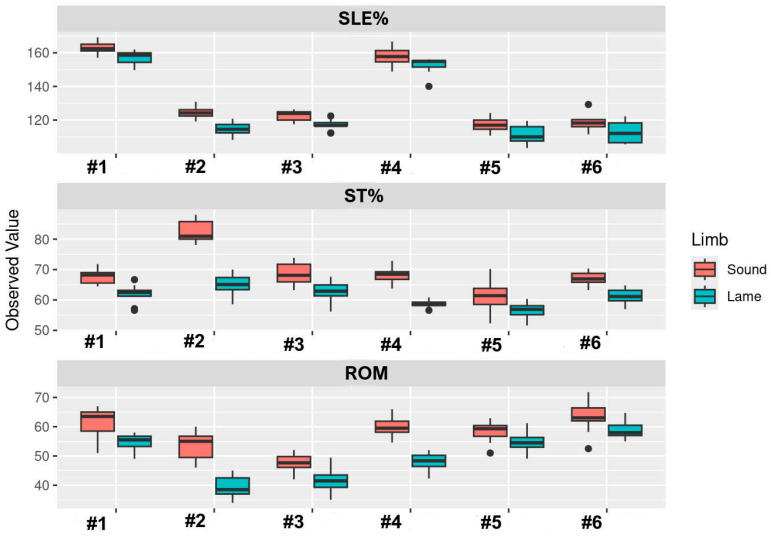
Distribution of SLE%, ST%, and ROM values for the sound limb and lame limb in the six dogs (#1–#6) included in the study.

Figure 4 shows the distribution of the observed values for each variable for each dog across the limbs of each dog (Figure 4).

The coefficients of variation (CV) and results of the Shapiro–Wilk normality test for each parameter and dog are presented in Table 3. The variability was generally low for SLE% and ST%, and moderate for ROM. The assumption of normality was not rejected (*p* > 0.05) for the vast majority of measurements, supporting the use of parametric methods for subsequent analysis.

### 3.3. Symmetry Indices and Inferential Statistics

The calculated symmetry indices (SI for SLE% and ST%, SA for ROM), along with their standard errors, 95% CIs, and *p*-values for the test against the 3% asymmetry threshold, are shown in Table 4. The observed asymmetry values confirmed the effectiveness of the lameness induction, with values ranging from 2.09% to 23.49%. However, for dogs with subtle lameness (SI/SA ≈ 3–5%), the CIs were wide and often included the 3% threshold, resulting in non-significant *p*-values (e.g., Dog #1 for SLE%, Dog #5 for ROM). This visualizes the challenge of detecting mild lameness with limited data.

### 3.4. Power and Sample-Size Estimation

Based on the observed coefficients of variation and using the delta-method framework, we estimated the number of valid trials required to achieve 80–90% statistical power for detecting an asymmetry exceeding 3%. For Stride Length (SLE%), assuming a mean CV of 3.5%, 29–39 trials per limb are required to achieve 80–90% power for detecting a true SI of 4% (Table 5). For Support Time (ST%), with a mean CV of 4.5%, 18–24 trials are needed (Table 6). For ROM, which exhibited a higher mean CV of 7%, fewer trials were required: 11–14 trials (Table 7).

For example, with 10 trials per limb, an asymmetry of 8 or more would be detected with 80% power.

Calculations were performed using the delta-method framework. CV = Coefficient of Variation, assumed to be equal for sound and lame limbs. The assumed mean CV for each parameter was 0.035 for SLE%, 0.045 for ST%, and 0.070 for ROM

## 4. Discussion

This study determined the number of trials required to detect true differences in SLE, ST, and ROM in a dog model of mild, induced forelimb lameness. Although the analyzed parameters are not classical primary indicators of lameness, they were sensitive enough to detect asymmetry associated with the induced mild lameness used in this study. By combining delta-method simulations with experimental data from six dogs, we demonstrated that sampling requirements are not universal but depend critically on the type of biomechanical variable. Our results directly challenge the adequacy of low trial numbers. For instance, while 10 trials provided 80% power only for detecting pronounced asymmetries (SI ≥ 8–10%), this number rose substantially for milder lameness. To detect a subtle asymmetry of 4% with 80% power, 29 to 251 trials were required, depending on the parameter (see Table 4, Table 5 and Table 6). This sharp increase in requisite trials, particularly for the common target of 3–5% asymmetry, is in stark contrast with the common practice of collecting three to five trials [14,23]. These results provide practical, parameter-specific guidelines for designing efficient gait studies and underscore the inadequacy of the commonly used five-trial protocol for detecting subtle lameness.

Importantly, similar mean values of symmetry indices may be obtained despite substantial differences in the variability of the underlying kinematic parameters. In the present study, the induced lameness resulted in modest shifts in mean values but increased stride-to-stride variability in the lame limb. This difference in variability directly influenced the width of the confidence intervals and, consequently, the number of strides required to reliably detect asymmetry. In this context, the marked inter-individual variability observed across dogs (Table 4) highlights that, even under standardized experimental conditions, asymmetry responses to mild induced lameness are not uniform. This variability is also evident in the stance time results at the 90% confidence level and directly contributes to the wide range of stride numbers required to achieve reliable detection of asymmetry.

To calculate mean symmetry data, some studies compute the mean of the symmetry indices from each trial, while others use the mean values of the trials as input data, calculating the index only once; since the final value is the same [31], we opted for the second method for its simplicity. The statistical foundation of our approach is the delta method, a widely used technique for deriving standard errors of functions of estimated parameters. The delta method employs a first-order Taylor series expansion to estimate standard error, an approximation that is most accurate when the function is locally linear—an assumption rarely violated in practice, and certainly not violated in our case [32]. By linearizing the symmetry index and symmetry angle, we obtained closed-form expressions for their asymptotic variance, which were then used to compute CIs and perform hypothesis tests. Our simulations confirmed that even with modest sample sizes (≈10 trials per limb), the delta method provided accurate estimates of standard error, validating its use in this context.

Determining an appropriate sample size is critical for ensuring that statistical inferences are both valid and efficient. General principles stipulate that sample size calculations must account for the desired significance level (α), power (1 − β), the expected outcome variability, and the minimum clinically relevant effect size [26,29,30]. Power analysis highlights the balance between Type I and Type II errors: selecting a lower α reduces the probability of false positives but may require a larger sample to maintain power, while increasing sample size reduces the risk of false negatives at the cost of resources [26,33]. Our sample size derivations adhere to these conventions, providing parameter-specific recommendations that balance statistical rigor with practical feasibility.

Accurate kinematic data depend on precise marker placement, given that misplacement and skin-movement artifacts can shift joint-angle waveforms vertically and alter extreme values [23,34]. To minimize this variability, we standardized the standing position during marker application, clipped the hair, used easily palpable bony landmarks, and ensured that the same experienced researcher placed all markers. Furthermore, all trials were recorded in a single session, thereby avoiding inter-session variability. Under these conditions, our data represent relative differences between limbs rather than absolute joint angles. Previous work has shown that two-dimensional video analysis provides accurate and repeatable sagittal joint motion [35], intraobserver repeatability is excellent [36] and that the influence of skin-movement artifacts on symmetry indices within a single session is minimal [34].

The classical criterion for diagnosing lameness has been a significant difference between limbs [3]. However, dogs exhibit a degree of natural asymmetry [9]; thus, small asymmetries (e.g., 3%) may not indicate true lameness. SI and SA were developed to quantify relative differences, with consensus cut-off values around 3% [37,38]. Lowering the threshold (e.g., to 2%) would reduce false negatives but risk misclassifying physiological asymmetry, as lameness and would require more trials [9]. In our simulations, five trials—a number often reported in kinematic studies—were clearly insufficient to detect mild asymmetries (SI ≈ 4–7%). A key insight is that the number of trials should be tailored to the variable measured. Angular measures such as ROM exhibited less variability and required fewer trials than linear or temporal measures, consistent with previous reports of parameter-specific variability [18,19,39]. The required number of trials is also influenced by the data collection protocol; for example, treadmill-based gait analysis, considered the gold standard in research settings, allows for the collection of many consecutive strides at a constant speed, leading to markedly reduced stride-to-stride variability and high within-subject repeatability after acclimation [40,41]; under those controlled conditions, fewer trials may be necessary to achieve reliable detection.

However, the methodology employed in our study—over-ground walking with manual video analysis—reflects a different, yet highly relevant, practical context. In many clinical, rehabilitative, and sporting environments (e.g., veterinary clinics, agility fields), access to sophisticated laboratory equipment is limited. Veterinarians assessing recovery from orthopedic surgery or trainers evaluating athletic performance often rely on these simpler, two-dimensional video analyses to obtain objective gait measures [36]. This approach provides an accessible and cost-effective method for longitudinal monitoring of an individual animal’s progress [3]. Our findings, therefore, provide essential guidance for these real-world applications, establishing evidence-based sample sizes for assessments conducted outside the gait laboratory. The statistical framework we present is equally valid for these scenarios, and our results demonstrate that increasing the number of recorded trials is a crucial strategy to compensate for the higher variability inherent in over-ground, non-consecutive stride collection, thereby improving the reliability of lameness detection in practical settings.

Although our study provides robust statistical guidelines, some limitations should be acknowledged. ROM was obtained from a single joint (the elbow). While elbow motion is representative of forelimb kinematics, further studies should examine other joints to confirm that similar sample sizes apply. We analyzed non-consecutive strides recorded over ground; although quadruped locomotion is highly repeatable [42], future studies based on treadmill acquisition and large numbers of consecutive strides could further explore the stability of asymmetry measures through subsampling approaches. Finally, dogs in this study had similar body dimensions; although we verified that changes in dog size produce proportional changes in the required sample size, broader sampling across breeds would strengthen generalization.

## 5. Conclusions

This study demonstrates that the common practice of collecting five trials is insufficient for the reliable kinematic detection of subtle lameness in dogs. The required number of trials is highly parameter-specific and varies dramatically with the magnitude of asymmetry. For example, detecting subtle lameness at a 3–4% threshold with high confidence (80–90% power) can require well over 100 trials for some parameters, far exceeding common practice, with minimum requirements ranging from as few as 11 to over 300 trials per limb.

To address this methodological gap, we developed a robust statistical framework based on the delta method. This framework provides researchers and clinicians with a practical tool for calculating CIs, performing hypothesis tests, and, most importantly, determining evidence-based sample sizes for gait studies tailored to their specific needs and acceptable error thresholds.

Although this pilot study focused on induced forelimb lameness, the proposed methodology is versatile and can be extended to larger datasets, other joints, and different lameness models. Future work should validate these results under treadmill conditions and in cases of more pronounced or bilateral lameness.

## Figures and Tables

**Table 1:** Descriptive characteristics of the dogs included in the study and experimental conditions.

Dog Number#	Breed	Sex	Age	Body Mass	BCS *
1	German Shorthaired Pointer	Male	3	28	4
2	Canarian Warren Hound	Male	7	24	4
3	Canarian Warren Hound	Female	5	22	4
4	Mixed	Female	5	25	5
5	English Pointer	Male	4	26	5
6	Weimaraner	Female	6	27	5

* According to the Association for Pet Obesity Prevention (https://www.petobesityprevention.org/surveys, accessed on 11 February 2026).

**Table 2:** Descriptive statistics of kinematic parameters for the sound limb (SL) and lame limb (LL) of the six dogs. Values are expressed as mean ± standard deviation (n = 10 trials per limb). *p*-values are from paired *t*-tests comparing SL and LL.

Variable	Id	SL N = 10	LL N = 10	*p*-Value
SLE%	#1	163.03 ± 3.77	157.16 ± 4.11	0.0038
	#2	124.48 ± 3.33	114.43 ± 4.03	<0.0001
	#3	122.82 ± 3.15	117.11 ± 3.16	0.0007
	#4	158.05 ± 5.29	152.52 ± 4.97	0.0269
	#5	117.20 ± 3.91	111.13 ± 5.83	0.0148
	#6	118.24 ± 5.02	112.52 ± 6.33	0.0388
ST%	#1	67.88 ± 2.53	61.95 ± 3.11	0.0002
	#2	82.38 ± 3.76	65.06 ± 3.52	<0.0001
	#3	68.38 ± 3.79	62.65 ± 3.89	0.0037
	#4	67.92 ± 2.71	58.62 ± 1.20	<0.0001
	#5	61.71 ± 5.44	56.51 ± 2.55	0.0172
	#6	67.08 ± 2.20	61.10 ± 2.48	<0.0001
ROM	#1	61.30 ± 5.19	54.60 ± 3.13	0.0033
	#2	53.70 ± 4.74	39.40 ± 3.69	<0.0001
	#3	47.56 ± 3.37	42.01 ± 4.54	0.0066
	#4	59.66 ± 3.49	48.16 ± 2.97	<0.0001
	#5	58.41 ± 3.64	54.69 ± 3.27	0.0274
	#6	63.23 ± 5.30	58.78 ± 3.10	0.0373

**Table 3:** Coefficients of variation (CV) and Shapiro–Wilk test results for normality for each kinematic parameter, dog, and limb.

		Coefficients of Variation		Shapiro–Wilk *p*-Values	
Variable	id	SL	LL	SL	LL
SLE%	#1	0.023	0.026	0.7583	0.1649
	#2	0.027	0.035	0.8600	0.8749
	#3	0.026	0.027	0.1950	0.3339
	#4	0.033	0.033	0.9096	0.0015
	#5	0.033	0.052	0.8798	0.3407
	#6	0.042	0.056	0.2712	0.1241
ST%	#1	0.037	0.050	0.4950	0.3636
	#2	0.046	0.054	0.1574	0.9468
	#3	0.055	0.062	0.4791	0.5198
	#4	0.040	0.020	0.6096	0.9073
	#5	0.088	0.045	0.6794	0.9821
	#6	0.033	0.041	0.9787	0.9107
ROM	#1	0.085	0.057	0.1424	0.2408
	#2	0.088	0.094	0.2264	0.6202
	#3	0.071	0.108	0.5066	0.5049
	#4	0.058	0.062	0.7713	0.6981
	#5	0.062	0.060	0.5444	0.9248
	#6	0.084	0.053	0.7776	0.4347

**Table 4:** Symmetry Index (SI) or Symmetry Angle (SA) values with standard errors, 95% CIs, and *p*-values for the test against the null hypothesis of asymmetry ≤ 3%.

Variable	id	SI	CI 95%	*p*
SLE%	#1	3.67 ± 1.10	(1.35; 5.98)	0.2770
	#2	8.41 ± 1.40	(5.48; 11.35)	0.0006
	#3	4.76 ± 1.18	(2.29; 7.24)	0.0755
	#4	3.56 ± 1.48	(0.46; 6.67)	0.3535
	#5	5.32 ± 1.96	(1.19; 9.44)	0.1267
	#6	4.96 ± 2.23	(0.28; 9.64)	0.1955
ST%	#1	9.14 ± 1.97	(4.99; 13.28)	0.0030
	#2	23.49 ± 2.21	(18.86; 28.13)	<0.0001
	#3	8.74 ± 2.63	(3.22; 14.25)	0.0212
	#4	14.71 ± 1.41	(11.74; 17.67)	<0.0001
	#5	8.79 ± 3.12	(2.23; 15.36)	0.0402
	#6	9.33 ± 1.64	(5.88; 12.79)	0.0006
ROM	#1	3.68 ± 1.02	(1.53; 5.82)	0.2584
	#2	9.70 ± 1.24	(7.11; 12.30)	<0.0001
	#3	3.94 ± 1.29	(1.23; 6.65)	0.2381
	#4	6.76 ± 0.84	(5.01; 8.52)	0.0001
	#5	2.09 ± 0.87	(0.27; 3.92)	0.8451
	#6	2.32 ± 0.99	(0.23; 4.41)	0.7484

**Table 5:** Sample size requirements for SLE%.

	Power								
SI	0.5	0.55	0.6	0.65	0.7	0.75	0.8	0.85	0.9
4	110	127	146	167	191	218	251	291	347
5	28	32	37	42	48	55	63	73	87
6	13	15	17	19	22	25	28	33	39
7	7	8	10	11	12	14	16	19	22
8	5	6	6	7	8	9	10	12	14
9	4	4	5	5	6	7	7	9	10
10	3	3	3	4	4	5	6	6	8
11	2	2	3	3	3	4	4	5	6
12	2	2	2	3	3	3	4	4	5

**Table 6:** Sample size requirements for ST%.

	Power								
SI	0.5	0.55	0.6	0.65	0.7	0.75	0.8	0.85	0.9
4	67	77	89	101	116	132	152	176	210
5	17	20	23	26	29	33	38	44	53
6	8	9	10	12	13	15	17	20	24
7	5	5	6	7	8	9	10	11	14
8	3	4	4	5	5	6	7	8	9
9	2	3	3	3	4	4	5	5	6
10	2	2	2	3	3	3	4	4	5
11	2	2	2	2	2	3	3	3	4
12	1	1	2	2	2	2	2	3	3

**Table 7:** Sample size requirements for ROM.

	Power								
SA	0.5	0.55	0.6	0.65	0.7	0.75	0.8	0.85	0.9
4	20	23	26	30	34	39	45	52	62
5	5	6	7	8	9	10	11	13	16
6	3	3	3	4	4	5	5	6	7
7	2	2	2	2	3	3	3	4	4
8	1	1	1	2	2	2	2	2	3
9	1	1	1	1	1	2	2	2	2
10	1	1	1	1	1	1	1	1	2
11	1	1	1	1	1	1	1	1	1
12	1	1	1	1	1	1	1	1	1

## Data Availability

The data presented in this study are available on request from the corresponding author.

## Abbreviations

The following abbreviations are used in this manuscript:

SD	Standard deviation
ICC	Intraclass correlation
SI	Symmetry index
SL	Sound limb
LL	Lame limb
SA	Symmetry angle
ROM	Angular range of motion
SLE	Stride length
ST	Stance time
CV	Coefficient of variation
CI	Confidence interval
